# Draft Genome Sequence of *Cupriavidus* UYMMa02A, a Novel Beta-Rhizobium Species

**DOI:** 10.1128/genomeA.01258-16

**Published:** 2016-11-10

**Authors:** Andrés Iriarte, Raúl Platero, Valeria Romero, Elena Fabiano, José R. Sotelo-Silveira

**Affiliations:** a Departamento de Bioquímica y Genómica Microbianas, IIBCE, Montevideo, Uruguay; b Departamento de Genómica, IIBCE, Montevideo, Uruguay

## Abstract

We present the draft genome of *Cupriavidus* UYMMa02A, a rhizobium strain isolated from root nodules of *Mimosa magentea*. The assembly has approximately 8.1 million bp with an average G+C of 64.1%. Symbiotic and metal-resistance genes were identified. The study of this genome will contribute to the understanding of rhizobial evolution.

## GENOME ANNOUNCEMENT

The *Cupriavidus* genus is comprised of Gram-negative, flagellated, rod-shaped bacteria belonging to the *Burkholderiaceae* family in the beta subclass of *Proteobacteria*. *Cupriavidus* bacteria are model organisms for the study of heavy-metal resistance (1–6), degradation of aromatic compounds (7), and synthesis of poly-hydroxybutyrate, making this genus an excellent candidate for biotechnological applications. Additionally, members of the *Betaproteobacteria* class are able to form nitrogen-fixing nodules in symbiotic associations with legumes (8, 9). Beta-rhizobia have been subsequently shown to colonize plant hosts worldwide with preference for the *Mimosoideae* subfamily (8–10). The first genome of beta-rhizobia, *Cupriavidus taiwanensis*, was published in 2008 (10).

Here, we report the draft genome sequence of the rhizobium *Cupriavidus* sp. strain UYMMa02A isolated from root nodules of *Mimosa magentea* naturally occurring in Uruguay. Interestingly, *Cupriavidus* sp. UYMMa02A is not related to the previously described symbiotic species *C. taiwanensis* or *C. necator* (11). The symbiotic bacterial isolation technique has been described elsewhere (11).

The strain was grown to stationary phase in TY media (12), harvested, and genomic DNA was isolated using the ZR fungal/bacterial DNA MiniPrep kit (Zymo Research). Sequences were generated using an Ion Torrent personal genome machine (PGM—Life Technologies), at the sequencing facility of the Institute of Biological, Research “Clemente Estable,” Montevideo, Uruguay. Standard procedure and manufacturer’s instructions were followed in each step, as described elsewhere (13). In brief, 400 base-pair libraries were prepared using the IonXpress Plus fragment library kit (Life Technologies, Inc.), followed by a Pippin Prep (Sage Science) size selection. Controls were performed by means of a high-sensitivity Bioanalyzer 2100 (Agilent) and Ion Sphere quality control kit (Life Technologies, Inc.). Sequencing was performed using an Ion PGM 400 sequencing kit (Life Technologies, Inc.).

A total of 1,394,358 high-quality reads were produced. *De novo* assembly was performed with SPADES assembler version 3.5.0 (14), using a preassembly approach with Mira version 4.0 (15). More than 98% of the generated reads, with an average length of 285 bp, were used in the assembly resulting in a mean nucleotide coverage of 341×. The final assembly has 8,184,499-bp length, distributed in 310 contigs. The *N*_50_ is 58,238, with a maximum contig length of 228,874 bp and a G+C content of 64.1%.

The genome was annotated using the RAST server with default search parameters (16, 17). As a result, 8,201 putative protein-coding sequences, 59 transfer RNA genes, and 17 rRNA genes were identified.

The draft genome of this beta-rhizobium strain will allow for testing of different hypotheses about the evolution of symbiosis in the genus as well as the study of the genetic basis of this complex and fundamental ecological process. A comparative genomic analysis will be included in future publications.

### Accession number(s).

The generated genome sequence has been deposited at GenBank under BioProject PRJNA306671. The version described in this paper is the first version under the accession no. LRMT00000000.

## References

[1] Goris J, De Vos P, Coenye T, Hoste B, Janssens D, Brim H, Diels L, Mergeay M, Kersters K, Vandamme P 2001 Classification of metal-resistant bacteria from industrial biotopes as *Ralstonia campinensis* sp. nov., *Ralstonia metallidurans* sp. nov. and *Ralstonia basilensis* Steinle et al. 1998 emend. Int J Syst Evol Microbiol 51:1773–1782. doi:10.1099/00207713-51-5-1773. 11594608

[2] Poehlein A, Kusian B, Friedrich B, Daniel R, Bowien B 2011 Complete genome sequence of the type strain *Cupriavidus necator* N-1. J Bacteriol 193:5017. doi:10.1128/JB.05660-11. 21742890 PMC3165677

[3] Hong KW, Thinagaran D, Gan HM, Yin WF, Chan KG 2012 Whole-genome sequence of *Cupriavidus* sp. strain BIS7, a heavy-metal-resistant bacterium. J Bacteriol 194:6324. doi:10.1128/JB.01608-12. 23115161 PMC3486403

[4] Li LG, Cai L, Zhang T 2013 Genome of *Cupriavidus* sp. HMR-1, a heavy metal-resistant bacterium. Genome Announc 1(1):e00202-12. doi:10.1128/genomeA.00202-12. PMC3569349 23409271

[5] Ray J, Waters RJ, Skerker JM, Kuehl JV, Price MN, Huang J, Chakraborty R, Arkin AP, Deutschbauer A 2015 Complete genome sequence of *Cupriavidus basilensis* 4G11, isolated from the Oak Ridge Field Research Center Site. Genome Announc 3(3):e00322-15. doi:10.1128/genomeA.00322-15. 25977418 PMC4432324

[6] Janssen PJ, Van Houdt R, Moors H, Monsieurs P, Morin N, Michaux A, Benotmane MA, Leys N, Vallaeys T, Lapidus A, Monchy S, Médigue C, Taghavi S, McCorkle S, Dunn J, van der Lelie D, Mergeay M 2010 The complete genome sequence of *Cupriavidus metallidurans* strain CH34, a master survivalist in harsh and anthropogenic environments. PLoS One 5:e10433. doi:10.1371/journal.pone.0010433. 20463976 PMC2864759

[7] Pérez-Pantoja D, De la Iglesia R, Pieper DH, González B 2008 Metabolic reconstruction of aromatic compounds degradation from the genome of the amazing pollutant-degrading bacterium *Cupriavidus necator* JMP134. FEMS Microbiol Rev 32:736–794. doi:10.1111/j.1574-6976.2008.00122.x. 18691224

[8] Chen WM, Laevens S, Lee TM, Coenye T, De Vos P, Mergeay M, Vandamme P 2001 *Ralstonia taiwanensis* sp. nov., isolated from root nodules of mimosa species and sputum of a cystic fibrosis patient. Int J Syst Evol Microbiol 51:1729–1735. doi:10.1099/00207713-51-5-1729. 11594603

[9] Moulin L, Munive A, Dreyfus B, Boivin-Masson C 2001 Nodulation of legumes by members of the beta-subclass of *Proteobacteria*. Nature 411:948–950. doi:10.1038/35082070. 11418858

[10] Amadou C, Pascal G, Mangenot S, Glew M, Bontemps C, Capela D, Carrère S, Cruveiller S, Dossat C, Lajus A, Marchetti M, Poinsot V, Rouy Z, Servin B, Saad M, Schenowitz C, Barbe V, Batut J, Médigue C, Masson-Boivin C 2008 Genome sequence of the beta-rhizobium *Cupriavidus taiwanensis* and comparative genomics of rhizobia. Genome Res 18:1472–1483. doi:10.1101/gr.076448.108. 18490699 PMC2527706

[11] Platero R, James EK, Rios C, Iriarte A, Sandes L, Zabaleta M, Battistoni F, Fabiano E 2016 Novel *Cupriavidus* strains isolated from root nodules of native Uruguayan mimosa species. Appl Environ Microbiol 82:3150–3164. doi:10.1128/AEM.04142-15. 26994087 PMC4959248

[12] Beringer JE 1974 R factor transfer in *Rhizobium leguminosarum*. Microbiology 84:188–198. doi:10.1099/00221287-84-1-188. 4612098

[13] Giorello FM, Romero V, Farias J, Scavone P, Umpiérrez A, Zunino P, Sotelo Silveira JR 2016 Draft genome sequence and gene annotation of the uropathogenic bacterium *Proteus mirabilis* Pr2921. Genome Announc 4(4):e00564-16. doi:10.1128/genomeA.00564-16. 27340058 PMC4919397

[14] Bankevich A, Nurk S, Antipov D, Gurevich AA, Dvorkin M, Kulikov AS, Lesin VM, Nikolenko SI, Pham S, Prjibelski AD, Pyshkin AV, Sirotkin AV, Vyahhi N, Tesler G, Alekseyev MA, Pevzner PA 2012 SPAdes: a new genome assembly algorithm and its applications to single-cell sequencing. J Comput Biol 19:455–477. doi:10.1089/cmb.2012.0021. 22506599 PMC3342519

[15] Chevreux B, Pfisterer T, Drescher B, Driesel AJ, Müller WE, Wetter T, Suhai S 2004 Using the miraEST assembler for reliable and automated mRNA transcript assembly and SNP detection in sequenced ESTs. Genome Res 14:1147–1159. doi:10.1101/gr.1917404. 15140833 PMC419793

[16] Aziz RK, Bartels D, Best AA, DeJongh M, Disz T, Edwards RA, Formsma K, Gerdes S, Glass EM, Kubal M, Meyer F, Olsen GJ, Olson R, Osterman AL, Overbeek RA, McNeil LK, Paarmann D, Paczian T, Parrello B, Pusch GD, Reich C, Stevens R, Vassieva O, Vonstein V, Wilke A, Zagnitko O 2008 The RAST server: Rapid Annotations using Subsystems Technology. BMC Genomics 9:75. doi:10.1186/1471-2164-9-75. 18261238 PMC2265698

[17] Overbeek R, Olson R, Pusch GD, Olsen GJ, Davis JJ, Disz T, Edwards RA, Gerdes S, Parrello B, Shukla M, Vonstein V, Wattam AR, Xia F, Stevens R 2014 The SEED and the Rapid Annotation of microbial genomes using Subsystems Technology (RAST). Nucleic Acids Res 42:D206–D214. doi:10.1093/nar/gkt1226. 24293654 PMC3965101

